# Prognostic significance of the CRAFITY score in hepatocellular carcinoma treated with immunotherapy: a systematic review and meta-analysis

**DOI:** 10.1186/s12885-023-10686-9

**Published:** 2023-03-13

**Authors:** Ming Yang, Yilin Pan, Wentao Wang

**Affiliations:** Department of Liver Surgery, West China Hospital, Sichuan University, 610000 Chengdu, P. R. China

**Keywords:** CRAFITY score, Liver cancer, Prognosis, Immunotherapy, Meta-analysis

## Abstract

**Background:**

This meta-analysis aimed to assess the performance of the CRAFITY (CRP and AFP in immunotherapy) score as a prognostic factor in hepatocellular carcinoma (HCC) treated with immunotherapy.

**Methods:**

The PubMed, Cochrane Library, and Web of Science databases were searched for published studies. Hazard ratios (HRs) with 95% confidence intervals (CIs) for overall survival (OS) and progression-free survival (PFS) outcomes were pooled using fixed- and random-effects models. Odds ratios (ORs) with 95% CI were used to measure the association of individual CRAFITY scores with the disease control rate (DCR).

**Results:**

Four eligible studies comprising 786 patients were included. The results indicate that a lower CRAFITY score is a significant predictor of better OS (HR = 0.22, 95% CI: 0.10–0.50) and PFS (HR = 0.36, 95% CI: 0.23–0.55) outcomes. In addition, the DCR was significantly higher in patients with lower CRAFITY scores (OR = 3.16, 95% CI: 2.00–4.99). A significant positive association between low CRAFITY scores and favorable prognoses was also observed in Barcelona Clinic Liver Cancer stage B/C/D patients.

**Conclusion:**

In this study, a low CRAFITY score was associated with better overall outcomes in HCC patients treated with immunotherapy. However, this finding requires further investigation.

## Introduction

Liver cancer remains a growing global health threat [1], and it is estimated that over 1 million patients will be affected annually by 2050 [2]. The 5-year survival rate for all stages combined is 18%. The poor outcome of hepatocellular carcinoma (HCC) patients results from late diagnosis and the refractory nature of the disease. Currently, liver cancer therapies consist of aggressive multimodal treatments, including surgery, transarterial chemoembolization, transarterial radioembolization, radiofrequency ablation, molecular targeted therapy, and immunotherapy [3–5]. Despite the advancement in comprehensive therapy and expected improvement in clinical outcomes, relapse, progression, and treatment failure remain frequent.

Immune checkpoint inhibitors (ICIs) are one of the most important classes of immunotherapy drugs that target negative regulatory proteins on T cells and enhance T-cell activation [6]. Previous studies have identified several predictive markers for immunotherapy responses derived from patient genomics data, such as noncoding and coding RNAs, DNA methylation, and mutational burden [7, 8]. However, there are currently no robust markers that predict clinical response.

Alpha-fetoprotein (AFP) is expressed in 70–80% of HCC patients, serving as a biomarker for diagnosis and surveillance [9]. C-reactive protein (CRP) is an acute-phase reactant protein synthesized by hepatocytes in response to inflammatory cytokines and is thought to be an important prognostic marker of liver cancer [10]. The combined use of serum AFP and CRP values (i.e., the CRAFITY score) has recently been recommended for identifying patients who will benefit from immunotherapy on the basis of the results of a retrospective multicenter study [11]. However, this study was retrospective, and synthetic evidence is lacking. Here, a systematic review and meta-analysis was performed to evaluate the significance of the combined use of AFP and CRP values in predicting the clinical outcomes of liver cancer patients treated with immunotherapy.

## Materials and methods

The Preferred Reporting Items for Systematic Reviews and Meta-Analyses statements were followed [12].

### Data sources and search

Two investigators conducted an independent literature search using the PubMed, Embase, Cochrane Library, and Web of Science databases from the databases’ inception to May 31, 2022 (Ming and Yilin). The following key terms were used: “Alpha-fetoprotein,” “C-reactive protein,” and “CRAFITY score.” The cited references of the relevant systematic reviews and conference proceedings were manually cross-checked to identify additional literature. Articles pertinent to liver cancer immunotherapy were selected for this meta-analysis. Searches were not restricted by language, country, or publication date. Articles were initially screened based on title and abstract reading, and then full texts of potentially relevant publications were obtained and reviewed by two authors independently (Ming and Yilin) to determine the publications’ eligibility. Any discrepancies were resolved by discussion, and a third assessor arbitrated any disagreements.

### Selection criteria

Eligible studies that conformed to the following criteria were included in this meta-analysis. (i) Hepatocellular carcinoma patients received ICIs. (ii) The pretreatment CRAFITY score comprising two indicators, AFP and CRP (0 points (AFP < 100 ng/mL and CRP < 1 mg/dL) indicating a low CRAFITY score, 1 point (either AFP ≥ 100 ng/mL or CRP ≥ 1 mg/dL) indicating intermediate, and 2 points (AFP ≥ 100 ng/mL and CRP ≥ 1 mg/dL) indicating high) was used as a prognostic factor. (iii) The main outcomes of interest were overall survival (OS) and progression-free survival (PFS). (iv) Tumor control or progression was defined according to radiological evaluations.

### Data extraction

Data were extracted from the retrieved full-text articles independently by two reviewers (Ming and Yilin). First, information from eligible publications was extracted, including the first author, study design, sample size, demographic features, clinicopathological characteristics, and immunotherapy regimens. Second, information about the following clinical data from included trials and their supplementary documentation was extracted, including the hazard ratio (HR) and 95% CI for OS and PFS and the number of patients with an antitumor response for calculating the disease control rate (DCR). Response Evaluation Criteria in Solid Tumors (RECIST) version 1.1 or modified RECIST (mRECIST) was used in the four included studies [11, 13–15]; the DCR was defined as the percentage of patients achieving complete or partial responses or with stable disease.

### Quality assessment

The Newcastle–Ottawa Scale [16] was used to assess study quality. The scale consists of three parameters: selection, comparability, and outcome assessment. The maximum possible score is 9 points, and studies with a score > 6 are regarded as high-quality.

### Statistical analysis

RevMan (version 5.3, Cochrane Collaboration) was applied to pool and analyze data. Pooled HR and 95% CI estimates for OS and PFS outcomes were obtained from each article, where possible. The odds ratios (OR) and 95% CI for the DCR were also retrieved from each article. If HRs with 95% CI were not reported, HRs with 95% CI were derived indirectly from the Kaplan–Meier curves using the methods described by Tierney [17]. Heterogeneity among studies was assessed using Cochran’s Q statistic and I^2^ statistics. Heterogeneity was considered statistically significant when I^2^ > 50% or *P* < 0.10. I^2^ values of 0–50%, 50–75%, and 75–100% represent low, moderate, and high heterogeneities, respectively. A fixed-effects model was used when heterogeneity existed; otherwise, a random-effects model was used.

## Results

### Identification of studies and study characteristics

**Fig. 1:**
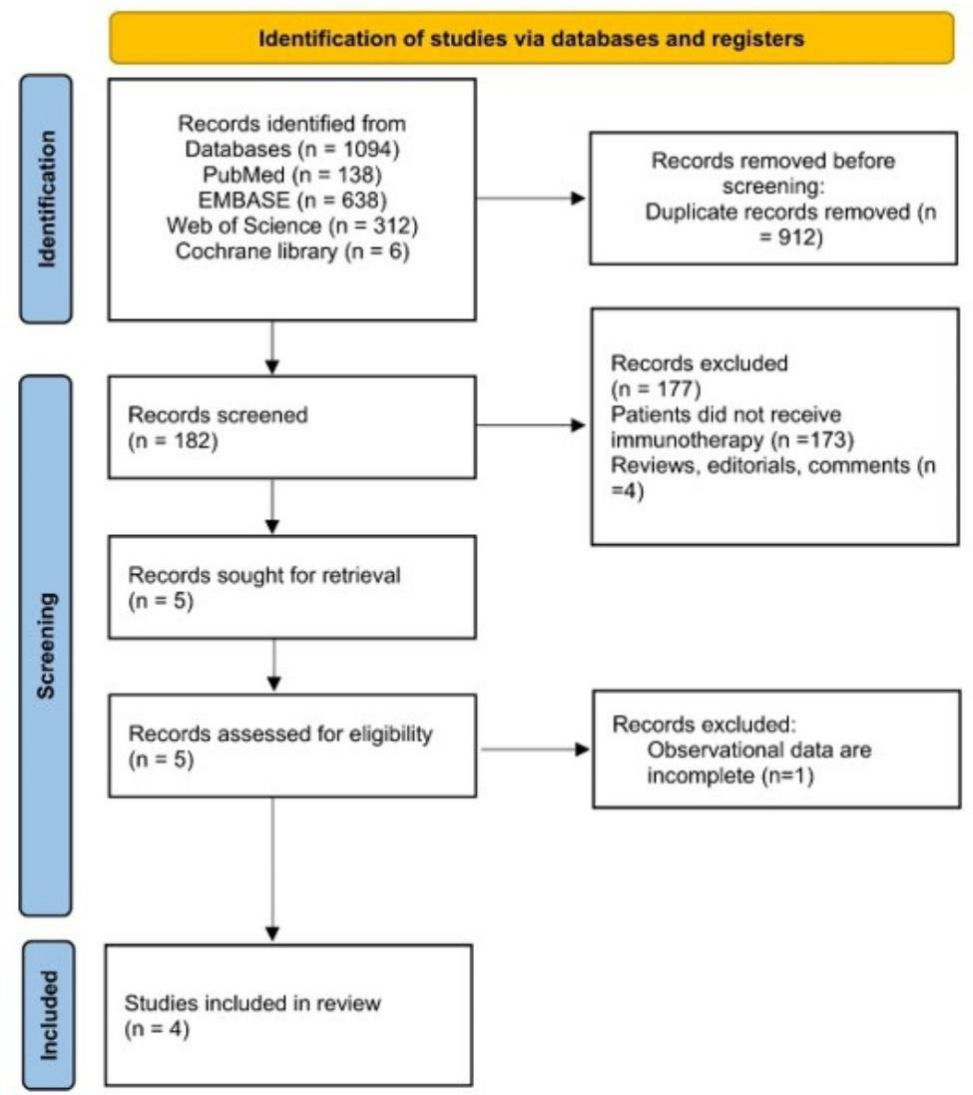
PRISMA diagram showing the identification of the eligible studies and reasons for exclusion

The Preferred Reporting Items for Systematic Reviews and Meta-Analyses flow diagram is presented in Fig. 1. The systematic search yielded 1,094 records through electronic searches of the PubMed (138 records), Embase (638 records), Web of Science (312 records), and Cochrane Library (6 records) databases. After duplicates were removed, 182 records remained. Based on the title and abstract, 177 irrelevant records were excluded. Four full-text articles were retrieved by two independent reviewers for further detailed assessment. One article was excluded [18] due to the lack of survival information. Eventually, four studies [11, 13–15] were included, comprising 786 hepatocellular carcinoma patients treated with immunotherapy. The overall quality of the four cohort studies [11, 13–15] was moderate; the Newcastle–Ottawa Scale scores ranged from 7 to 8.

Four studies [11, 13–15] investigated HCC patients undergoing immunotherapy; immunotherapeutic regimens included anti-programmed death (ligand) 1 (anti-PD-(L)1)-based immunotherapy monotherapy plus bevacizumab/ramucirumab/TKI and anti-CTL-4/anti-CD38. Three studies [13–15] investigated patients with Barcelona Clinic Liver Cancer (BCLC) stage B or later, with only one study [11] including patients with BCLC stage A. In particular, Scheiner et al. reported treatment data for two independent cohorts of HCC patients. A summary of the baseline patient information is presented in Table 1.

**Table 1:** Patient demographics and baseline characteristics

	Scheiner et al., n = 292		Yang et al., n = 108	Hatanaka et al., n = 297	Teng et al., n = 89
	Training set, n = 190	Validation set, n = 102			
Age, years	66.2 ± 10.4	64.6 ± 11.9	57[44.0, 69.0]	73.0[68.0, 78.0]	61.3[56.4,67.8]
Male, n (%)	153(81%)	83(81%)	96(89%)	243(82%)	75(84%)
Etiology					
Viral	55(29%)	39(38%)	95(88%)	149(50%)	79(89%)
Non-viral	135 (71%)	63 (62%)	13(12%)	148(50%)	10(11%)
Child-Pugh A/B/C	101/72/17	72/28/2	-	279/-/-	76/13/-
ECOG PS					
0	88(46%)	46(45%)	-	238(80%)	35(39%)
≥ 1	102(54%)	56(55%)	-	59(20%)	54(61%)
Lines of systemic therapy					
Front line	82(43%)	35(34%)	-	169(57%)	49(55%)
later line	108(57%)	67(66%)	-	128(43%)	40(45%)
BCLC stage A/B/C/D	2/21/149/18	-/12/88/2	-/24/82/-	17/121/155/4	-/23/66/-
Patients with complete CRAFITY score					
0	53(28%)	18(32%)	25(23%)	147(49%)	-
≥ 1	137(72%)	38(68%)	83(73%)	150(51%)	-
Type of immunotherapy-based regimen administered	Anti-PD-(L)1 monotherapy or plus bevacizumab/TKI/ramucirumab	Anti-PD-(L)1 monotherapy or plus bevacizumab/TKI/anti-CTL4-4/anti-CD38	Anti-PD-(L)1 plus lenvatinib	Atezolizumab plus bevacizumab	Atezolizumab plus bevacizumab
NOS score	8		7	7	8

Abbreviations: BCLC, Barcelona Clinic Liver Cancer; ECOG, Eastern Cooperative Oncology Group; CRAFITY, C-reactive protein and α-fetoprotein in immunotherapy; CTLA-4, cytotoxic T-lymphocyte-associated protein 4; PD-(L)1, programmed cell death protein 1/programmed cell death 1 ligand 1; TKI, tyrosine kinase inhibitor; NOS, Newcastle-Ottawa Scale, -Data were missing

### Predicted effect of CRAFITY score on OS Outcomes among HCC patients treated with immunotherapy

Each of these four studies [11, 13–15] reported survival data and assessed the predicted effects of the CRAFITY score on OS outcomes among HCC patients. OS was defined as the time from the start of immunotherapy until the date of death or last follow-up in the four publications [11, 13–15].

**Fig. 2:**
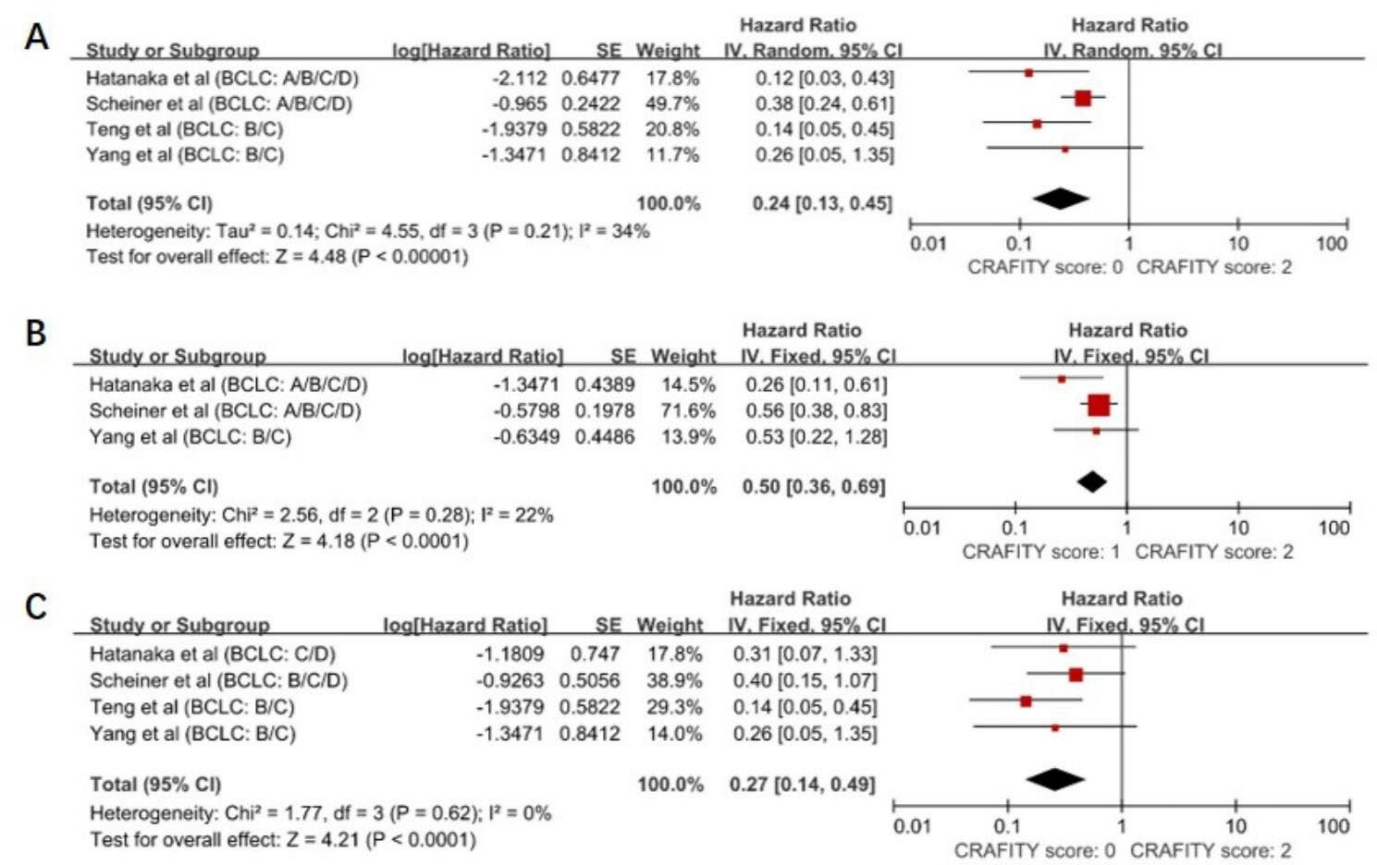
Forest plot depicting improved overall survival outcomes with low and intermediate CRAFITY scores in the subgroup analysis according to BCLC stage. (A) The OS outcomes of BCLC A/B/C/D patients with CRAFITY score: 0. (B) The OS outcomes of BCLC A/B/C/D patients with CRAFITY score: 1. (C) The OS outcomes of BCLC B/C/D patients with CRAFITY score: 0

A random-effects model was used to pool the included studies [11, 13–15] and demonstrated that patients with a low CRAFITY score (0 points) had better OS outcomes than those with a high CRAFITY score (2 points) (HR = 0.24, 95% CI: 0.13–0.45, *P* < 0.00001), with a moderate heterogeneity between studies (I^2^ = 34%, *P* = 0.21) (Fig. 2A).

Analyses of patients pooled for intermediate (1 point) and high (2 points) CRAFITY scores showed similar results. An intermediate CRAFITY score was also significantly associated with a better prognosis (HR = 0.50, 95% CI: 0.36–0.69, *P* < 0.0001). The meta-analysis approach showed low heterogeneity (I^2^ = 22%, *P* = 0.28). (Fig. 2B). Considerable heterogeneity is observed in the aforementioned results. A stratified analysis according to the BCLC staging demonstrated a significant association between low CRAFITY scores and better OS outcomes among BCLC stage B/C/D patients treated with immunotherapy (HR = 0.27, 95% CI: 0.14–0.52, *P* < 0.0001), with no heterogeneity (I^2^ = 0%, *P* = 0.41) (Fig. 2C).

### Predicted effect of the CRAFITY score on PFS outcomes among HCC patients treated with immunotherapy

Hatanaka et al. [13] and Teng et al. [14] reported the PFS outcomes of patients with liver cancer after immunotherapy. In these two publications [13, 14], PFS was defined as the time from the initial immunotherapy until radiological disease progression or death.

**Fig. 3:**
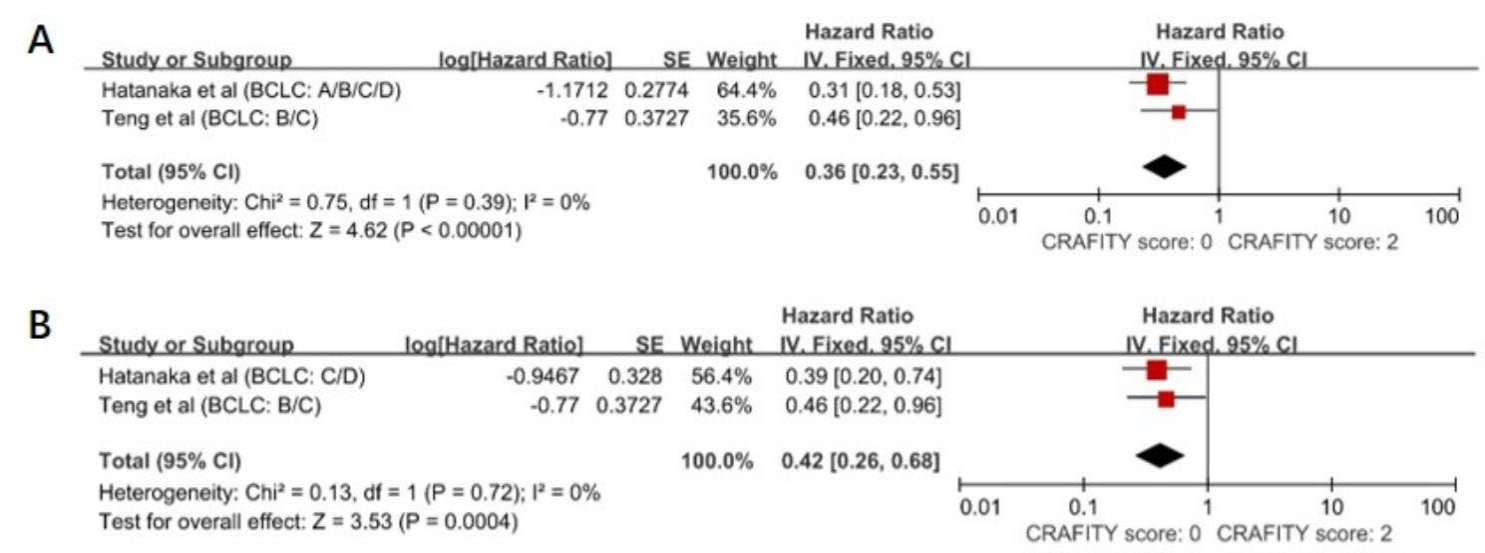
Forest plot depicting improved progression-free survival outcomes among patients with low CRAFITY scores in the subgroup analysis according to BCLC stage. (A) The PFS outcomes of BCLC stage A/B/C/D patients. (B) The PFS outcomes of BCLC stage B/C/D patients

The pooled data indicated that a low CRAFITY score was associated with increased PFS rates in HCC patients treated with immunotherapy with a pooled HR estimate of 0.36 (95% CI: 0.23–0.55; Fig. 3A), without any heterogeneity (I^2^ = 0%, *P* = 0.39). Next, a subgroup analysis was performed according to the BCLC staging system. As shown in Fig. 3B, BCLC stage B/C/D patients with low CRAFITY scores had better PFS outcomes after receiving immunotherapy (HR = 0.42, 95% CI: 0.26–0.68, *P* = 0.0004), with no heterogeneity (I^2^ = 0%, *P* = 0.72).

### The CRAFITY score predicts radiological response in HCC patients treated with immunotherapy

Liver cancer patient responses to immunotherapy were determined by radiologic monitoring (RECIST/mRECIST) in all four publications [11, 13–15]. The difference in responses to immunotherapy among the patients with 0 (CRAFITY-low), 1 (CRAFITY-intermediate), and 2 points (CRAFITY-high) was investigated.

**Fig. 4:**
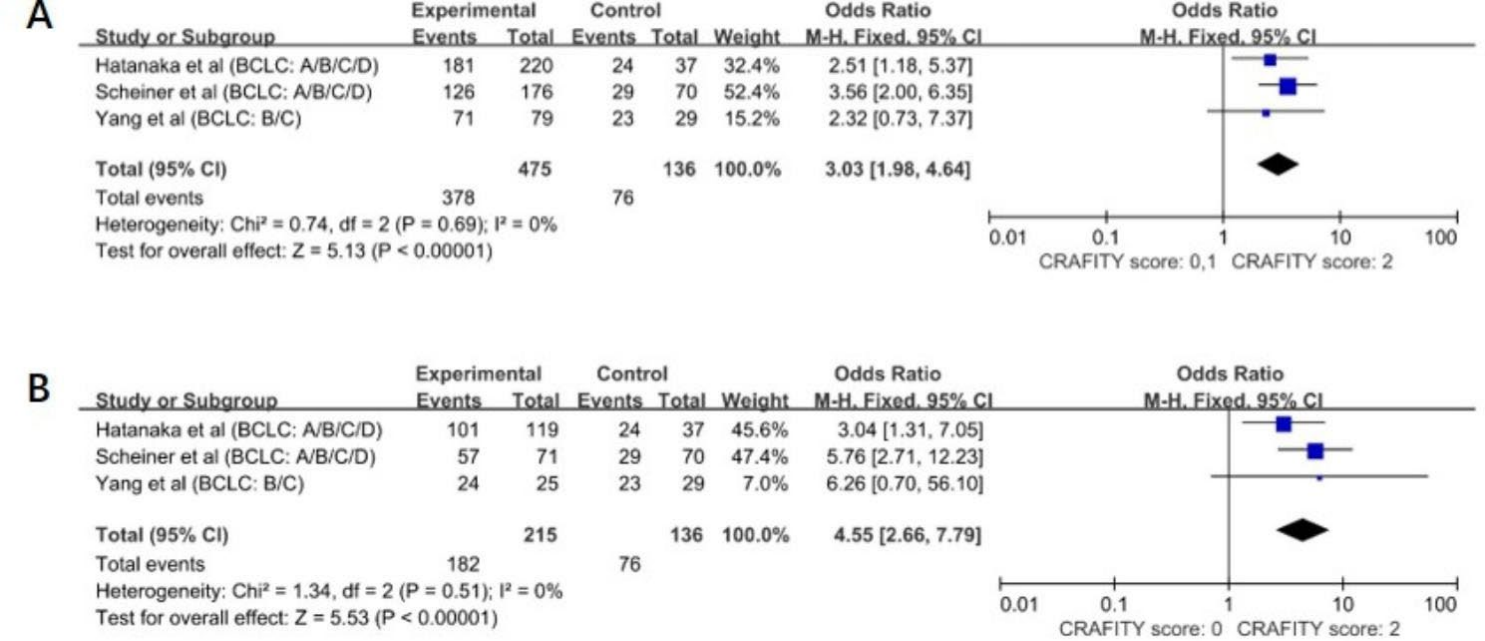
Forest plot depicting the disease control rate in the subgroup analysis according to CRAFITY score. (A)The DCR of patients with CRAFITY score: 0,1. (B) The DCR of patients with CRAFITY score: 0

The pooled result showed a significantly increased DCR in the CRAFITY-low and CRAFITY-intermediate groups (OR = 3.03, 95% CI: 1.98–4.64, *P* < 0.00001), with no heterogeneity (I^2^ = 0%, *P* = 0.69) (Fig. 4A) compared with that in the CRAFITY-high group. In addition, the CRAFITY-low group also showed a higher DCR than the CRAFITY-high group (OR = 4.55, 95% CI: 2.66–7.79, *P* < 0.00001), with no heterogeneity (I^2^ = 0%, *P* = 0.51) (Fig. 4B).

## Discussion

The current estimates indicate that liver cancer is the seventh most common cancer and the fourth main cause of cancer-related death worldwide [19]. Over half of liver cancer patients are diagnosed with advanced-stage disease. Nevertheless, systemic therapy has been shown to improve local and systemic liver cancer control [5, 20]. The clinical use of ICIs is rapidly expanding; atezolizumab plus bevacizumab was recently approved as first-line therapy for unresectable, advanced HCC [21]. Although immunotherapy has improved the prognosis of patients with advanced liver cancer, it is important to note that only a subset of patients benefits from this intervention [22]. To the best of our knowledge, there is no uniform biomarker to predict liver tumor responses to ICIs. In this study, we specifically sought to identify a validated clinical predictor of positive outcomes for ICI.

Recently, Scheiner et al. proposed using the CRAFITY score to predict the treatment response and survival of patients with liver cancer receiving immunotherapy with PD-(L)1 antibodies [11]. However, more evidence is needed to confirm the predictive ability of the CRAFITY score in immunotherapy. In this meta-analysis, we first assessed the relationship between the CRAFITY score and patient prognosis. Overall, we included four trials enrolling 786 HCC patients treated with immunotherapy. The results of the current study demonstrated that patients with a low CRAFITY score had better OS and DFS outcomes high DCRs. In BCLC stage A/B/C/D patients, the pooled HRs of OS outcomes were moderately heterogeneous. Taking this further, the OS of patients with CRAFITY score 1 was also longer than those with CRAFITY score 2. A stratified analysis by the BCLC staging (BCLC B/C/D) showed remarkably decreased heterogeneity, but the prognostic significance was not reduced. ICI failure as monotherapy in a phase III trial for advanced HCC was recently reported [23]. However, another phase III randomized trial found that the endpoint OS outcomes were significantly improved by atezolizumab plus bevacizumab compared with sorafenib [24]. Furthermore, systemic administration of checkpoint blockade can result in immune-related adverse events (irAEs) [25]. Despite combining immunotherapy and targeted therapy, approximately half of liver cancer patients do not respond to ICIs. A reliable clinical marker will enable the selection of patients maximally responsive to immunotherapy and reduce the application of therapy to patients who are unlikely to benefit.

AFP is an important serological indicator of HCC that was previously identified in human fetal serum [26]. To date, the AFP level is recommended for routine screening, diagnosis, and prognostic stratification of liver cancer [9, 27, 28]. AFP levels are also used to identify patients with liver tumors who are suitable for liver transplantation [29]. CRP is now considered to have prognostic value in patients with cancer independent of tumor stage. A recent review discussed the in-depth link between traditional circulating inflammatory markers, such as CRP, IL-6, and systemic inflammation in cancer patients [30]. As one of the hallmarks of cancer, cancer-associated inflammation is an important event in tumor progression and may affect the tumor microenvironment. Chronic inflammation is a key inducer of the immunosuppressive microenvironment. A previous study revealed the lymphocyte-to-monocyte ratio to be a positive prognostic factor in colorectal cancer patients with liver metastasis after radiofrequency ablation [31]. Zhang et al. found that patients with high CRP levels have shorter PFS times than those with low CRP after PD-1 inhibitor treatment [32]. Therefore, our meta-analysis specifically focused on whether combining AFP and CRP values can serve as a biomarker for immunotherapy. In the current analysis, all included trials assessed survival via the CRAFITY score, and tumor immunotherapy produced better therapeutic effects in patients with a low CRAFITY score.

The response evaluation was performed radiologically according to RECIST version 1.1 or mRECIST in the four included studies; a high DCR was significantly associated with low and intermediate CRAFITY scores. Furthermore, the CRAFITY score system is not a risk prediction model, as it does not require expert computational skills to calculate patient data. Thus, the CRAFITY score might be a practical, effective tool for stratifying liver cancer patients and enhance the response rate of ICI.

This study has several limitations. First, only four good-quality retrospective studies from six countries (Austria, Germany, Italy, Switzerland, China and Japan) were included after an exhaustive systematic search. Prospective randomized studies and a large sample size are needed before this CRAFITY score can be routinely recommended. Second, two studies used atezolizumab plus bevacizumab, one used anti-PD-(L)1 plus lenvatinib and one used anti-PD-(L)1 monotherapy or a combination with targeted treatment. Third, the different BCLC stages of the patients is a limitation and differences between stage B, C, and D patients may be obscured. To fully understand the effects of tumor burden, sufficient data for prespecified subgroup analyses are required.

## Conclusion

In this meta-analysis, the CRAFITY score was sufficient to distinguish outcome differences among patients treated with immunotherapy. Low and intermediate CRAFITY scores were associated with a lower risk of death and increased response rates to immunotherapy among HCC patients. Therefore, these findings provide some evidence for the clinical applicability of the CRAFITY score.

## Data Availability

All data generated or analyzed during this study are included in this published article.

## Abbreviations

CRAFITY score: CRP and AFP in immunotherapy score
HRs: Hazard ratios
CI: Confidence interval
PFS: Progression-free survival
OS: Overall survival
ORs: Odds ratios
BCLC: Barcelona Clinic Liver Cancer
HCC: Hepatocellular carcinoma
PRISMA: Preferred Reporting Items for Systematic Reviews and Meta-Analyses
WOS: Web of Science
CRP: C-reactive protein
AFP: Alpha-fetoprotein
DCR: Disease control rate
RECIST: Response Evaluation Criteria in Solid Tumors
